# Reflections on Clinical and Statistical Use of the Penetration-Aspiration Scale

**DOI:** 10.1007/s00455-017-9809-z

**Published:** 2017-05-22

**Authors:** Catriona M. Steele, Karen Grace-Martin

**Affiliations:** 1 Toronto Rehabilitation Institute, University Health Network, 550 University Avenue, 12th Floor, Toronto, ON M5G 2A2 Canada; 2 Rehabilitation Sciences Institute, University of Toronto, 500 University Avenue, Suite 160, Toronto, ON M5G 1V7 Canada; 3 The Analysis Factor, 430 W State St, Suite #204, Ithaca, NY 14850 USA

**Keywords:** Deglutition, Deglutition disorders, Dysphagia, Penetration-aspiration, Videofluoroscopy, Statistics

## Abstract

The 8-point Penetration-Aspiration Scale (PAS) was introduced to the field of dysphagia in 1996 and has become the standard method used by both clinicians and researchers to describe and measure the severity of airway invasion during swallowing. In this article, we review the properties of the scale and explore what has been learned over 20 years of use regarding the construct validity, ordinality, intervality, score distribution, and sensitivity of the PAS to change. We propose that a categorical revision of the PAS into four levels of increasing physiological severity would be appropriate. The article concludes with a discussion of common errors made in the statistical analysis of the PAS, proposing that frequency distributions and ordinal logistic regression approaches are most appropriate given the properties of the scale. A hypothetical dataset is included to illustrate both the problems and strengths of different statistical approaches.

## Introduction

In 1996, Rosenbek and colleagues published a now seminal article introducing the 8-point Penetration-Aspiration Scale (PAS) [1]. This scale was developed to characterize the severity of airway invasion events viewed during videofluoroscopy, capturing the location to which material is observed to travel and then qualifying that information based on whether material remains there at the end of the swallow or has been ejected to safer (anatomically higher) locations. The scale has become widely used as an industry standard for the interpretation of videofluoroscopy and has also been adapted for interpreting the fiberoptic endoscopic examination of swallowing (FEES) [2–5]. In recognition of the fact that 20 years have passed since of the introduction of this scale, we present this article reflecting on both the clinical and statistical uses of the scale. An example data set is included for tutorial purposes in the discussion of statistical analysis of the PAS. Although we have done our best to provide balanced comments, readers should be aware that the comments in this article reflect the opinions of the authors, and others may have differing points of view. In this respect, we hope to stimulate discussion and debate.

## Purpose of the Penetration-Aspiration Scale (PAS)

In the words of the original scale developers, the PAS was designed with the primary purpose of describing and providing a means of quantifying the severity of penetration and aspiration events. The scale was also described to be “a potentially powerful outcome measure for clinical trials designed to investigate the efficacy of various swallowing treatments… [and possibly] equally useful to the clinician interested in demonstrating a functional change in the individual patient.” [1] p. 97. However, the authors signaled caution about the statistical characteristics of the scale and how these might need to be handled in research applications. In particular, the authors acknowledged that further experiments would be needed to confirm whether the PAS had ordinal and interval qualities [6]. An ordinal scale is one for which each increasing score on the scale is understood to represent greater severity than the previous level. An interval scale is one in which the data lie along a continuum and the spacing between adjacent levels is considered to be equal [7]. Many studies of dysphagia severity and treatment outcome have treated the PAS as ordinal or even interval, and have used parametric statistics such as ANOVA to compare measures of central tendency across groups or difference scores either across groups or within groups over time (e.g., [8–14]). In other studies, the scale has been reduced to a binary variable or treated as categorical (e.g., [15–22]). In this article, we review issues of construct validity, ordinality, score distribution, scale reduction, and sensitivity to change. We also discuss issues related to the protocols and instruments that are used to collect data regarding penetration and aspiration and how constraints inherent to these measurement methods may impact the validity of PAS scores.

## Construct Validity

Penetration and aspiration are understood to be serious events for people with dysphagia, and are widely considered to represent a risk for respiratory sequelae such as pneumonia [23, 24]. The ability to grade the relative severity of an airway invasion event during swallowing assessment holds great clinical value and is also important in research. There are, of course, a variety of ways in which this challenge can be approached. The PAS primarily adopts the approach of capturing the anatomical depth to which material travels: a score of 1 reflects no entry of material into the airway, scores of 2–5 reflect penetration of material past the laryngeal additus into the supraglottic space and traveling as far as the true vocal folds, while scores of 6–8 reflect tracheal aspiration of material below the true vocal folds (see Table 1). A retrospective case series reported by Pikus and colleagues lends credence to the idea that divisions of airway invasion by depth (i.e., PAS level 1 vs levels 2–5, levels 6–7 and level 8) are associated with relative risk for pneumonia [23]. However, other studies suggest that the relationship is not that straight forward [25]. It is well accepted in clinical circles that not every patient with tracheobronchial aspiration (whether sensate or silent) will progress to developing pneumonia [24, 26]. Factors that are widely thought to be relevant for pneumonia pathogenesis or other adverse consequences include: whether the aspirated material is expelled effectively from the larynx [23] or cleared over the next few hours [27]; the volume of material aspirated [28]; the bacterial and chemical composition of the material aspirated [29–31]; and the general health status and immune response of the patient, including factors such as advanced age, oral health, dependence for oral care and feeding, use of multiple medications, ambulatory versus bed-bound status, and use of tube feeding [25, 26, 32–35].

**Table 1:** 8-point Penetration-Aspiration Scale, developed by Rosenbek and colleagues (1996) [72]

1. Material does not enter the airway
2. Material enters the airway, remains above the vocal folds, and is ejected from the airway
3. Material enters the airway, remains above the vocal folds, and is not ejected from the airway
4. Material enters the airway, contacts the vocal folds, and is ejected from the airway
5. Material enters the airway, contacts the vocal folds, and is not ejected from the airway
6. Material enters the airway, passes below the vocal folds, and is ejected into the larynx or out of the airway
7. Material enters the airway, passes below the vocal folds, and is not ejected from the trachea despite effort
8. Material enters the airway, passes below the vocal folds, and no effort is made to eject

To their credit, the authors of the PAS acknowledged in the original manuscript that the scale deals *only* with the depth of airway invasion and the associated phenomenon of “ejection,” namely whether or not the material or is cleared via biomechanical or volitional mechanisms to safer locations (i.e., from below the true vocal folds to the supraglottic space, or from the supraglottic space into the pharynx). No claims were made regarding the estimation of aspirant volume, nor does the scale capture the timing of airway invasion relative to the onset or completion of the swallow. Consequently, labeling airway invasion events using the 8-points on the PAS *does not* discriminate between different mechanisms responsible for aspiration such as premature escape of material from the mouth into the pharynx, delayed laryngeal vestibule closure, or the spillover of residue into the larynx after the swallow.

## Physiological Interpretations of the PAS

In our opinion, rating the severity of penetration and aspiration according to the depth of airway invasion not only allows for detailed description but also allows the clinician to draw inferences regarding the sensory and motor integrity of different regions of the pharynx and larynx. Such inferences should never be made on the basis of a single bolus, or even a single volume or consistency. Rather, an astute clinician should be watching for patterns and considering what the pattern of presentation suggests about the integrity of both the sensory and motor components of swallowing function. Videofluoroscopy observations should, of course, also be interpreted in conjunction with other information gained about the patient in the course of the preceding clinical examinations. And, inferences regarding either sensory or motor integrity may need additional investigations for confirmation. The following paragraphs synthesize physiological knowledge that may be useful to consider when thinking about different PAS scores.

In healthy swallowing, structural movements leading to closure of the laryngeal vestibule are expected to begin prior to the bolus reaching the entry to the airway [36–38]. It is therefore considered abnormal for a bolus to enter the laryngeal vestibule prior to, or during the swallow. The only exception to this statement occurs in the case of a PAS score of 2 (sometimes referred to as “high” or “flash” penetration), in which a portion of the bolus briefly enters the upper section of the laryngeal vestibule, but is then ejected back into the pharynx. This ejection typically occurs as the result of biomechanical events such that movement of the arytenoid process toward the undersurface of the epiglottis squeezes the penetrated material back towards the laryngeal additus and pharynx, without any conscious or volitional action on the part of the person. This phenomenon, which was originally categorized by Rosenbek and colleagues to fall within the category of penetration, has subsequently been shown to occur in healthy individuals and is no longer considered abnormal [11, 39].

The laryngeal vestibule is densely populated with afferent receptors for the superior laryngeal nerve [40–42]. Excitation of the internal branch of the superior laryngeal nerve (iSLN) via electrical stimulation is one of the most effective methods for eliciting a swallow response in animal models [43]. It follows that when the sensory and motor function of the larynx is intact, the entry of foreign material into the supraglottic space can reasonably be expected to serve as a physiologic stimulus that will trigger an immediate swallow response with laryngeal vestibule closure, thereby enabling the biomechanical squeezing of penetrated material back out of the larynx [41, 44, 45]. Levels 2 and 4 on the PAS are both illustrative of this phenomenon, and they both capture successful clearance of penetrated material out of the laryngeal vestibule. Although scores of 2 are now known to occur in healthy swallowing [11, 39], readers may not be aware that scores of 4 are rarely observed [1, 3]. Penetration into the supraglottic space that does not trigger an immediate swallow response with effective clearance of the penetrant (i.e.,PAS levels 3 and 5) should be considered abnormal and should bring into question the integrity of the reflexive sensory-motor responses that are usually initiated with excitation of iSLN receptors [46–48].

With deeper degrees of airway invasion, the bolus crosses the true vocal folds to the trachea and reaches a new afferent nerve territory, namely that of the recurrent laryngeal nerve. The excitation of RLN receptors is expected to trigger reflexive laryngeal adduction and cough responses [42], ideally expelling the invading material back above the vocal folds and out of the airway. The different levels of aspiration on the PAS capture the effectiveness of this expected cough response. Interestingly, the original version of the PAS was a 9-point scale, and included an extra level which discriminated between ejection from beneath the vocal folds into the supraglottic space and ejection from beneath the vocal folds past the supraglottic space and out of the airway. However, the distribution of scores in the original dataset, which comprised 75 thin liquid boluses from 15 stroke patients with dysphagia, did not contain a single example of ejection from beneath the vocal folds completely out of the airway [1]. The two subglottal ejection possibilities were, therefore, collapsed into level 6, and the wording was modified to capture ejection of material, *either* into the supraglottic space, or out of the airway. Interestingly, even with this modification, ejection from beneath the vocal folds into the supraglottic space was only seen for 8 boluses (3%) in the original dataset [1]. Several subsequent studies continue to suggest that level 6 is an exceptionally rare PAS score [3, 49–51].

Level 7 on the PAS captures the scenario in which material falls beneath the true vocal folds and an ejection response is attempted by the patient, but there is no expulsion of the material. Despite the ineffective ejection attempt, spontaneous attempts to cough or throat clear by the patient are considered to reflect sensory integrity below the vocal folds. Level 8, which is referred to as silent aspiration, represents failure of both sensory and motor components of the expected response: there is no apparent awareness of material in the tracheo-bronchial tree and no attempt on the part of the patient to initiate expulsion of material from the trachea.

## Rare Scores

As mentioned previously, scores of 4 and 6 appear to be much less common than the other scores on the PAS. For researchers, the rarity of these scores poses challenges in terms of score distribution. In a clinical context, although the scale has important descriptive value, the relative rarity of these levels raises interesting questions about whether they are genuinely distinguishable from similar or adjacent levels on the scale, and whether the descriptive differences associated with these scores are clinically important. A related question for which data are not readily available in the literature is to ask about trends in inter-rater disagreement across the different levels of the scale. It would be very interesting to understand, for example, whether a putative score of 4 is more commonly “mis-scored” as a 3, a 5 or perhaps a 2 or a 6? A better understanding of the PAS levels that are more prone to differences of opinion across raters, and what the difference patterns are, would provide useful material for debate about possible scale revisions.

## Ordinality

Two years after the original publication of the PAS, McCullough et al. [6] published the results of a clinician survey in which the relative severity of each PAS level was ranked when paired with the other levels from the scale. Survey responses actually showed that clinicians were uncertain how to rank the severity of levels 3 and 5, in which material reaches and remains in the supraglottic space or on the true vocal folds without ejection. A majority (87%) of survey respondents indicated that they considered level 3 to be more severe than level 4, in which there is effective ejection of penetrated material into the pharynx. Similarly, 71% of respondents ranked level 5 as more severe than level 6, in which there is effective ejection of aspirated material into the supraglottic space (or, hypothetically, into the pharynx). Furthermore, 58% of respondents ranked level 3 (with no ejection from the supraglottic space) as more severe than level 6 (which includes the hypothetical possibility of ejection into the pharynx). Despite these responses, the authors concluded that the scale displayed *essentially* ordinal properties and argued that respondents might not have had sufficient opportunity to develop comfort with the scale, given its novelty. To the best of our knowledge, there has never been a subsequent survey to determine whether opinions regarding ordinality of the scale have evolved. Nevertheless, it is striking to us that the opinions reflected in the McCullough et al. [6] survey response patterns are consistent with the physiological concepts outlined in the previous section. We propose that the apparent lack of ordinality implies a need to re-organize and reconceptualize the PAS as a categorical scale (“Categorical PAS”), with four categories of progressively more severe impairment, which we will label alphabetically for clarity. This proposed reconceptualization of the PAS is illustrated in Table 2. Readers should be cautioned that this proposed Categorical PAS has not undergone validation.

**Table 2:** Proposed reorganization of the 8-point Penetration-Aspiration Scale into a 4-level Categorical Penetration-Aspiration Scale

Categorical PAS level	Original PAS scores	Description
A	1, 2, and 4	PAS levels 1 and 2 reflect normal function. Similarly, PAS level 4 reflects an effective response to the slightly deeper penetration of material into the supraglottic space, resulting in the absence of any material in the airway at the end of the swallow
B	3, 5, and 6	PAS Levels 3, 5, and 6 all capture abnormal situations in which material remains in the laryngeal vestibule at the end of the swallow, extending as deep as (but not below) the level of the true vocal folds. These levels reflect failure of supraglottic levels of airway protection. Furthermore, unless timely attempts to initiate secondary clearing swallows are seen, these levels on the PAS may also reflect some degree of iSLN impairment
C	7	PAS Level 7 reflects failure of supraglottic, glottal and tracheal airway protection mechanisms in the presence of some residual recurrent laryngeal nerve sensory integrity
D	8	PAS Level 8 reflects impairment both of effective cough responses to aspiration and also of the sensory circuits that are typically expected to trigger protective cough reflexes

## Intervality

Based on the frequency of severity rankings for paired levels of the PAS obtained in their survey study, McCullough et al. [6] were also able to explore the extent to which the scale displayed interval properties by constructing a derived scale of *z*-scores. With the exception of levels 5 and 6, the derived scale maintained the expected structure of lower *z*-scores for lower numbered levels on the scale. However, the spacing between levels was acknowledged not to be equal, ranging from *z*-score values as small as 0.19 between levels 3 and 4 to values as large as 1.65 between levels 6 and 7, and displaying mean spacing of 0.79 with a standard deviation of 0.5. Strictly speaking, these results fail to support assumptions of intervality.

The levels of the PAS are labeled numerically with discrete integer values. It is important to recognize that decimal places have no interpretable meeting on this scale. Nevertheless, throughout the dysphagia literature, it is commonplace to find parametric measures of PAS central tendency reported for groups of patients or research participants, with significant digits up to two decimal places (e.g., [8, 9, 12, 13, 52]). This practice has two major drawbacks. First, to report mean PAS scores in this fashion runs the risk of representing the airway protection capability of a group of individuals using a score that rarely or never happens. This would appear to be the case in an example reported recently by Pearson et al. [13] who describe a group of patients with a mean PAS score of 4.42. Secondly, the specification of decimal places encourages scientists to treat the scale as continuous in parametric statistics and to interpret small differences between groups as potentially both statistically and clinically significant. A recent, but certainly not unique example in this regard can be found in a manuscript by Langmore et al. [8] in which PAS scores were compared between head and neck cancer patients randomized to receiving neuromuscular electrical stimulation treatment vs a sham intervention. At the post-treatment examination, group mean PAS scores were reported as 5.1 (±1.8) and 4.9 (±2.1), respectively. Although removal of the decimal places from these scores would place both groups at an identical PAS score level of 5, and the authors themselves note that group differences in PAS scores < 1 have marginal clinical significance, the analysis in that study identified the group difference as being statistically significant [8].

## Statistical Analysis of the Penetration-Aspiration Scale

There is much debate in the field of statistics and measurement about whether and when it is appropriate to treat ordinal variables as interval and continuous. In their textbook, *Understanding Advanced Statistical Methods* [53], Westfall & Henning advise, “One answer is that the better the discrete data fill the continuum, the better the continuous model is as an approximation” (p. 40) and offer the following rule of thumb: “If the set of possible discrete outcomes is 10 or more, then a continuous model may provide an adequate approximation to the distribution of the discrete random variable” (p. 40). (We note here that with 8 levels, the PAS would not satisfy this criterion.)

It is important to distinguish between ordinal variables that are discrete measurements of a numerical, continuous, unmeasurable latent variable, such as Likert scale variables, and those that contain qualitative differences among categories that cannot be distinguished numerically [7]. Even interval level variables can have qualitative meaning within a specific research context. For example, consider a variable such as the number of days out of the past 30 in which a patient was hospitalized. While the number of days is unequivocally ratio under any measurement system, in the research context of understanding what happened to a patient, there is a qualitative difference between 0 and 1 days of hospitalization. In our opinion, the lack of available evidence to confirm either ordinality or intervality of the PAS means that it would be more appropriate to use frequency measures to represent the typical or most common patterns of airway invasion seen in a group of patients.

## Repeated Measures Considerations

One of the most interesting challenges that clinicians and researchers face, when characterizing a person’s swallowing safety, is the fact that scores may vary within a person across a series of swallows. It is expected (and desirable from a treatment planning perspective) that swallowing safety may vary across different bolus volumes [5], consistencies [54–57], postural maneuvers [58–60] and even across more subtle factors such as barium concentration [61]. However, the literature also suggests that penetration-aspiration events are unlikely to occur consistently within a person when the same task is repeated several times (for example, repeated 5 ml boluses of thin liquid) [15]. For this reason, it is generally suggested that an adequate challenge of swallowing safety should include several repetitions of a task and standard protocols (either for clinical or research purposes) provide a means of ensuring an equal number of opportunities to elicit or observe the problem as a basis for comparison. It is common practice for the worst score seen across a protocol to be used to represent the patient’s status; however, this risks skewing the overall impression towards one of impairment [15, 49, 50]. An alternative approach advocated by Martin-Harris and colleagues in the MBSImp protocol is to treat the first thin liquid bolus in a videofluoroscopy as a warm-up trial, and not to factor this bolus into the representation of a person’s swallowing safety status [49]; thus, patients who manage to implement spontaneous compensatory techniques that improve swallowing safety after an initial problem would not be unduly penalized for the initial swallow and would not be classified as having penetration or aspiration. When data are grouped at the participant level based on evidence of at least one aspiration event, the fact that aspiration is not constant contributes variation that makes it more difficult to appreciate distinct pathophysiological mechanisms behind impaired swallowing safety [15, 50].

There are several alternatives available to the approach of using the worst score to represent a person’s swallowing safety. One of these is to sum PAS scores across a fixed number of repetitions for a cumulative PAS score, which would reflect the frequency of scores of concern; however, because it is also common to terminate a protocol for safety reasons when several examples of aspiration have been observed, it may be difficult to guarantee that all patients will be able to complete the expected number of task repetitions and rules for scoring non-completed boluses due to safety bailout would need to be clearly specified. Using mean and median PAS scores to represent an individual’s swallowing safety status for a particular task has similar drawbacks to using worst scores; these scores may be questionably representative of the patient’s pattern of airway protection. Consider, for example, two hypothetical patients who present with the following PAS scores across 5 thin liquid boluses:

- Patient A (8, 5, 3, 5, 2); and
- Patient B (7, 5, 3, 7, 1).

Although the worst PAS scores are close for both patients (8 and 7, respectively), the fact that Patient A displays silent aspiration might (or might not) reflect a more serious concern from a clinical perspective. On the other hand, the occurrence of a score of 7 on two occasions in Patient B might be considered more serious than the single occurrence of a score of 8 on Patient A’s initial swallow, depending on clinical circumstances. Both patients would appear identical if mean (4.6) or median (5) values were used to capture their airway protection status. Given that scores are known to vary within individuals across repeated trials, we suggest that for clinical purposes, the most informative way to represent PAS scores may be to report *both* the mode and the worst score across a set of swallows. In the current example, this would result in scores of 5–8 and 7–7 for the two patients, respectively.

## Number of Levels

As mentioned previously, survey research by McCullough et al. [6] suggested that clinicians were not in full agreement regarding the relative severity of different levels on the PAS, particularly for scores in the penetration range (2–5). One solution to the lack of clarity distinguishing the different levels is to reduce the scale into fewer levels. This approach has been adopted quite commonly in research [15–22, 62] and it has the further potential advantage of limiting the opportunity for differences of opinion to arise between raters. Indeed, a study by Hind and colleagues [63] showed that agreement between novice and experienced raters was better when matching within a 3-score range (i.e., 1 point above or below the expert reference score) compared to looking for exact matches. Of course, evidence of improved inter-rater agreement related to fewer forced-choice rating options, by itself, is not an adequate justification for scale reduction. We propose that decisions to transform the 8-point PAS into fewer levels should be guided both by a physiological framework and by an understanding of trends in scoring by raters. Standardisation in protocols, data acquisition methods, and rating procedures and rules are paramount to control for other factors that may contribute to differences in rating decisions across clinicians or researchers.

## Constraints Associated with Assessment Methods and Procedures

In this regard, it is important to note that constraints inherent to different methods of acquiring swallowing data may limit the degree to which PAS scores can be considered accurate and reliable. These constraints are not limitations of the PAS itself, but rather of the contexts in which the data are acquired or gathered. Recognition of these constraints also provides opportunities for procedural standardisation that may improve the reliability of fluoroscopy ratings. Three examples of this type of constraint are described below:

1. As mentioned above, the literature suggests that scores of 4 and 6 in which there is successful ejection of material from a more serious to a less serious location rarely occur. However, instructions for use of the PAS provide no guidance regarding the length of time over which the clinician should watch for this ejection. Anecdotal experience from the first author’s research suggests that people with dysphagia frequently perform more than one swallow for a bolus, and that deep penetration or aspiration on an initial or early subswallow for a given bolus may evolve across later clearing swallows of the same bolus, either improving or deteriorating. In this circumstance, it becomes challenging to know whether to score swallowing safety based on the worst subswallow in the series or the ultimate status at the end of the series. Similarly, it is widely presumed that the presence of residue in the valleculae or pyriform sinuses at the end of a swallow represents a risk for post-swallow aspiration [64]; however, our ability to capture the true danger associated with residue depends on the length of post-swallow surveillance. Radiation exposure concerns further limit this surveillance opportunity. Current instructions regarding use of the PAS do not provide guidance on this point.
2. A recent study by Bonilha and colleagues [65] explored agreement for PAS ratings between two different versions of the same videofluoroscopy recordings with different temporal resolutions. The results showed that penetration-aspiration events that were detected in recordings with 30 images per second were missed 20% of the time when those same recordings were rendered at a lower resolution of 15 images per second. Thus, it appears that penetration-aspiration events may sometimes be very brief and are prone to being missed by raters. It follows that our ability to accurately detect the disposition and evolution of an aspiration event from a worst to better or best to worse score may also be susceptible to variation based on the temporal resolution of a videofluoroscopy recording. This is analogous to the often-quoted philosophical question about whether a tree falling unwitnessed in the forest makes a sound. Our ability to accurately detect airway invasion events is limited by both the temporal resolution and the duration of the methods used to seek evidence of the problem.
3. A similar scenario is appreciable when one considers using the PAS scale in the context of endoscopic rather than videofluoroscopic examinations of swallowing. A well-known constraint of endoscopy is the fact that a brief period of white-out obscures visibility of the pharynx and airway during the swallow [66]. When residual material is seen in the larynx or trachea after the swallow, it is logical to infer that penetration-aspiration has occurred. However, it is impossible for the clinician to rule out the possibility that material entered the larynx or trachea during the white-out period but then disappeared from view (either through ejection into the pharynx or by traveling deeper into the trachea). Thus, it seems implausible that PAS scores indicating ejection (i.e., 2, 4 and 6) could be validly or reliably detected using endoscopy. The susceptibility of particular PAS scores to being missed provides further reason to consider reducing the complexity of the scale to a smaller number of levels. The categorical reconceptualization of the PAS proposed in Table 2 should be less susceptible to this concern, given that the scores are grouped based on status/appearance at the end of the swallow.

## Options for Analysis

The remainder of this article is a tutorial for researchers on statistical approaches to analyzing penetration-aspiration data. For illustration purposes, we will use a hypothetical data set. Let us assume that this dataset contains data for 80 patients enrolled in a dysphagia rehabilitation trial based on pre-treatment evidence of at least one PAS score ≥3 for both thin liquid and mildly thick liquid boluses on a standard videofluoroscopy assessment involving up to 3 boluses of each consistency. We recognize that some readers may consider this situation to represent a rather narrow snapshot of patient performance, but this is done in the interests of illustration and clarity. Let us further assume that these individuals were randomly assigned either to an experimental group or a control group. The primary research question for this illustrative dataset is to determine whether swallowing safety has improved in the experimental group, based on a post-treatment videofluoroscopy assessment including the same thin and mildly thick liquid swallowing tasks. The appendix contains data showing worst PAS scores for each bolus type at the outcome assessment by patient. We acknowledge the possibility that using worst scores involves the previously identified possibility of bias towards impairment.

### Quantitative Approach

As mentioned above, many articles in the dysphagia literature have treated the PAS scale as a continuous quantitative scale with presumed interval properties. Table 3 illustrates the descriptive statistics that would be obtained if this approach were applied to the hypothetical dataset, for example in a general linear model ANOVA.

**Table 3:** Sample descriptive statistics for the hypothetical data set by treatment group and consistency, if the PAS is treated as an interval scale

Group	Consistency	Mean	Standard error	95% CI lower bound	95% CI upper bound
Control	Thin	3.40	0.37	2.66	4.14
	Mildly thick	3.40	0.37	2.66	4.14
Experimental	Thin	3.18	0.37	2.44	2.76
	Mildly thick	2.03	0.37	1.29	2.76

**Fig. 1:**
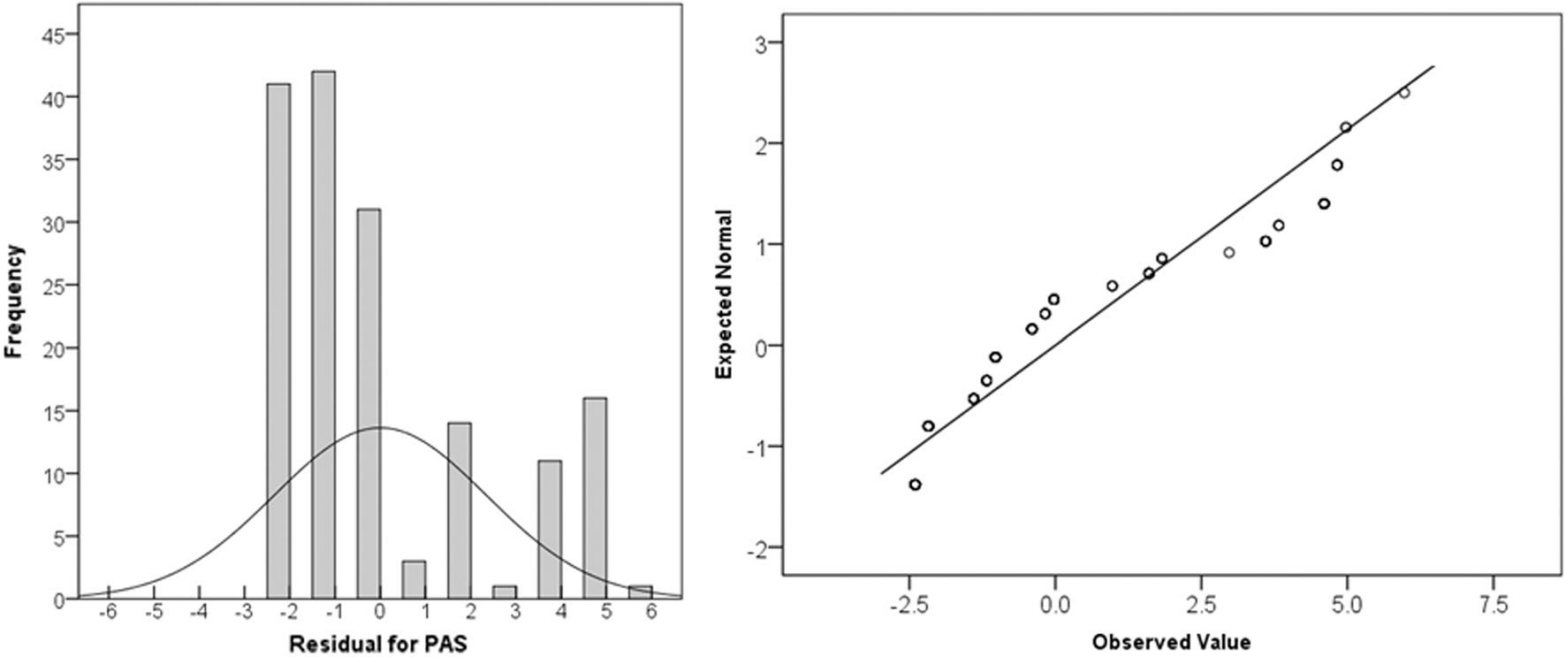
Distribution and quantile plot of the residuals for PAS scores in the hypothetical dataset

Figure 1 shows the distribution of residuals for these data. The distribution is clearly skewed, violating the assumption of normal distribution for residuals. Although linear models are robust to departures from normality, the skew that is observed in this case means that readers should question the credibility and accuracy of *p* values in both ANOVA or ordinary least squares regression models. Checking for normality in residual distribution is an important step in determining whether or not linear statistical models can be used for interpreting a dataset of PAS scores. Given evidence that certain scores are extremely rare, we speculate that the majority of PAS datasets will fail to show normal residual distribution.

### Ordinal and Categorical Approaches

If treating the PAS as quantitative violates model assumptions and is not best-practice, what is the alternative? The usual answer for ordinal data is to use non-parametric statistics. While these are still a good option in very simple studies, their limitations make them unsuitable as an all-around tool. Most non-parametric tests are based on rank statistics. Common rank-based statistics include the Wilcoxon Signed Rank Test, Mann–Whitney *U* Test, and Spearman correlations [67]. These tests work well for ordinal outcome variables and should be a strong consideration for the PAS when the design and research questions allow. Their disadvantage, though, is a big one: they are tests, not models [68]. Most research questions require more sophisticated analysis than simple group comparisons. Non-parametric statistics cannot handle interactions, covariates, or more than the simplest repeated measures. Although it is tempting to turn instead to statistical models designed for quantitative data, despite the inability of the PAS to meet their assumptions, there are better alternatives. In the following section, we will discuss descriptive and modeling options for categorical data that test all necessary hypotheses without making untenable assumptions.

#### Descriptive Statistics

Medians are usually suggested as the descriptive statistic of choice for ordinal data [7]. However, although they represent the center of the distribution well, unlike means, their robustness to the shape of the distribution is also their biggest disadvantage. For variables like the PAS, with only a few possible discrete values and an often highly skewed distribution, differences in scores above or below a median may not be reflected in median differences. Rather than using an inappropriate and uninterpretable statistic like the mean, however, we suggest using one of the following. While all require more than a single value to describe the location of the distribution, we contend that the increase in meaning is worth the added complexity.

##### Frequency Tables or Graphs

**Fig. 2:**
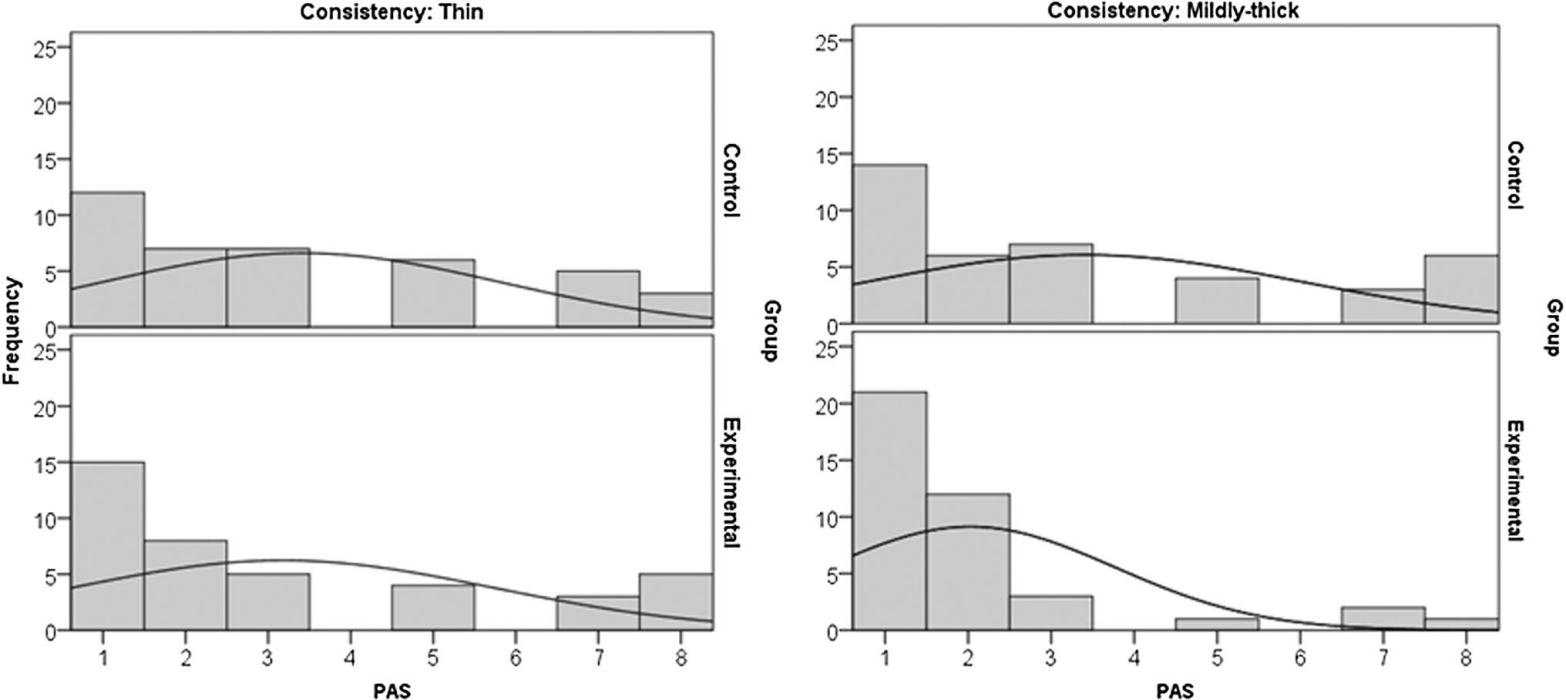
*Bar charts* showing the frequency distribution of the different PAS levels in the hypothetical dataset by treatment group and consistency

Table 4 shows the counts and percent frequencies of each PAS score for each treatment group by bolus consistency (thin or mildly thick). Figure 2 illustrates the frequency distributions of these data using bar charts. Frequency tables have the advantage of being appropriate whether we are using the full 8 levels on the PAS scale as nominal or collapsing scores into categories that preserve meaningful order. The patterns are clear in the illustrative dataset. First, PAS values of 4 and 6 do not occur at any point. For both consistencies, the experimental group has a higher percentage frequency of “healthy” PAS scores of 1 and 2, and a corresponding lower frequency of “scores of concern” (i.e., scores of 3 and higher, represented by the gray-shaded columns in the table). The appearance of better outcomes in the experimental group is more noticeable for the mildly thick liquid consistency than for the thin consistency. The greatest difference in score frequency between groups is seen for PAS scores of 8 (i.e., silent aspiration) on mildly thick liquids, which has a frequency of only 2.5% in the experimental group versus 15% in the control group.

**Table 4:** Frequency counts and percentages for each PAS score in the hypothetical data set, by treatment group and consistency

Group	Consistency	Statistic	PAS = 1	PAS = 2	PAS = 3	PAS = 4	PAS = 5	PAS = 6	PAS = 7	PAS = 8
Control	Thin	Count	12	7	7	0	6	0	5	3
		%	30.0%	17.5%	17.5%	0.0%	15.0%	0.0%	12.5%	7.5%
	Mildly thick	Count	14	6	7	0	4	0	3	6
		%	35.0%	15.0%	17.5%	0.0%	10.0%	0.0%	7.5%	15.0%
Experimental	Thin	Count	15	8	5	0	4	0	3	5
		%	37.5%	20.0%	12.5%	0.0%	10.0%	0.0%	7.5%	12.5%
	Mildly thick	Count	21	12	3	0	1	0	2	1
		%	52.5%	30.0%	7.5%	0.0%	2.5%	0.0%	5.0%	2.5%

##### Quantiles

Another method that is commonly used to summarize frequency distribution is the five-number summary, consisting of the minimum, 25th percentile, median, 75th percentile, and maximum. Table 5 provides a quantile summary for the illustrative data set. In this case, it is clear that the minimum, 25th percentile, and median PAS scores are all shifted towards the healthy end of the scale for both groups, regardless of bolus consistency. Maximum scores, however, remain stable at a PAS score of 8. Summarizing frequency data by quantile has the advantage of being familiar to most researchers (taught in every introductory statistics course), easy to interpret, meaningful, and appropriate. However, for scales such as the PAS, this method only works if one can establish ordinal categories. There will certainly be data sets and research contexts where this is too crude a measure to understand the subtleties in the data. Another option for those situations is to report deciles.

**Table 5:** PAS quantile scores for the hypothetical dataset by treatment group and consistency

Group	Consistency	Minimum	25th percentile	Median	75th percentile	Maximum
Control	Thin	1	1	3	5	8
	Mildly thick	1	1	2.5	5	8
Experimental	Thin	1	1	2	5	8
	Mildly thick	1	1	1	2	8

#### Models for Analyzing Nominal Multicategory Data

In this section, we will discuss several different types of logistic regression (binary, multinomial, and ordinal) as approaches that are suitable for analyzing the PAS provided that it is treated as a categorical variable. A major advantage of using logistic regression models (compared to the non-parametric rank statistic approaches mentioned previously) is that repeated measures, covariates, interactions, and quadratic effects can all be easily included.

It is assumed that the reader will already be familiar with ordinary linear regression, in which a continuous and unbounded dependent variable with interval or ratio properties, *Y*, is modeled as a function of different levels of a predictor variable, *X*_1_ *, X*_2_ *, X*_3_, …*X*_k_. The equation for linear regression is written as follows:

$$Y_{i} = \beta _{0} + \beta _{1} X_{{1i}} + \beta _{2} X_{{2i}} + \ldots + \beta _{k} X_{{ki}} + \varepsilon _{i}.$$

The beta functions in this equation reflect the intercept,* β*_0_, which is the average value of *Y* when all values of *X* = 0, and the coefficients* β*_1_ *X*_1i_,* β*_2_ *X*_2i_, etc., which must be added to the intercept to reach the values of *Y* for each unit of the predictor, *X*. The function* ε*_I_ at the end of the equation is the error term in the model.

Logistic regression is similar to linear regression, except that the dependent variable is no longer a continuous parameter with interval or ratio properties, but rather the natural log-odds of a particular value of a categorical parameter occurring. The odds ratio is calculated as the probability (Pr) of a given categorical value, *P*, divided by the inverse probability of that categorical value (1−*P*). This can be easily illustrated using the example of a binary version of the PAS comparing “healthy” scores of 1 and 2 to scores of concern (≥3). Here, the odds ratio for obtaining a score of concern would be written as

$${\text{Odds (PAS }} \ge { 3) = }\frac{{\Pr \;({\text{PAS }} \ge { 3})}}{{\Pr \;({\text{PAS < 3}})}} = \frac{\Pr \;(P)}{\Pr \;(1 - P)}.$$

An odds ratio of 1 would reflect an equal probability of obtaining a score from either category. Odds ratios >1 reflect a greater probability of obtaining a score in the category of interest (in this case, PAS ≥ 3), whereas odds ratios <1 reflect a greater probability of obtaining a score in the comparison or reference category (in this case, PAS < 3). For the interested reader, additional information regarding odds ratios, as well as both linear and logistic regression, can be accessed through the free webinar series at www.analysisfactor.com.

##### Binary Logistic Regression

If the PAS is reduced to 2 categories (i.e., healthy vs unhealthy), binary logistic regression will usually be the best option for analysis. The equation for binary logistic regression is written as follows:

$${\text{Ln}}\left( {{\text{Odds}}} \right){\mkern 1mu} = \beta _{0} + \beta _{1} X_{1} + \beta _{2} X_{2} + {\mkern 1mu} \ldots {\mkern 1mu} + \beta _{{kXk}} + \varepsilon _{i}.$$

Rather than using ordinary least squares, logistic regression explores the estimated values of all parameters in an iterative fashion until it finds the most likely value for the model (i.e., Maximum Likelihood Estimation). The outcome is expressed in the log-odds of a specific response category, with each *β* coefficient reflecting the difference in log-odds. The exponent of the log-odds value is an odds ratio, such that the *β* exponent reflects the amount by which the odds ratio for the dependent variable category needs to be multiplied for each one-unit change in the predictor variable, *X*.

##### Multinomial Logistic Regression

Although binary models are common and familiar to most readers, many clinicians and scientists may feel that reduction of the PAS to a two-category variable sacrifices important levels of detail. Multinomial logistic models can be used for situations in which the dependent variable has 3 or more categories; these are essentially a set of binary models [69, 70], which provide detailed estimates of treatment effect and a separate set of coefficients for each categorical response option in comparison to one category that is treated as the reference category. In principle, this could be achieved by running several parallel binary logistic regression models; however, the multinomial approach provides a more accurate result based on the inclusion of a single error variance term. The process is as follows:

- The *dependent* variable is coded into multiple binary 1/0 variables for each outcome category except one (the reference category). There will be M-1 binary outcome variables for M categories. The reference category is assigned a value of 0 for each of these binary variables.
- The multinomial logistic regression then estimates a separate binary logistic regression model for each of those binary variables. The result is M-1 binary logistic regression models. Each one measures the effect of the independent variables on the log-odds of that outcome category, in comparison to the reference category. Each model has its own intercept and regression coefficients—the predictors can affect each category differently.
- The multinomial logistic regression equation, with the subscript *h* indicating each category of the dependent variable, is written as follows:

$${\text{Ln}}\left( {{\text{Odds}}_{h} } \right){\mkern 1mu} = \beta _{{0h}} + \beta _{{1h}} X_{1} + \beta _{{2h}} X_{2} + {\mkern 1mu} \ldots {\mkern 1mu} + \beta _{{kh}} X_{k} + \varepsilon _{i}.$$

The main disadvantage of this approach (i.e., complexity) is also its main advantage (i.e., detail). There are a few consequences of this complexity. The first is that there are many coefficients to interpret, which can make patterns difficult to see. Secondly, when the independent variables are also categorical, every combination of outcome category and independent variable category must be present in the data in order for the model to compute. In the illustrative dataset, there are no cases of PAS = 4 or 6. This issue, which is called zero cell counts, will cause a failure of model convergence if these values occur in one condition but not another (e.g., pre-treatment but not post-treatment). A work-around for the current data set would be to recode the PAS levels that are not missing data as purely nominal or categorical variables: *A* = 1, *B* = 2, *C* = 3, *D* = 5, *E* = 7 and *F* = 8. In a multinomial logistic regression model, the odds of each of these 6 possible outcome categories for the PAS would be compared using a reference category of PAS = 1 (i.e., category A). The effect of each predictor (treatment group and consistency) would be estimated separately for each outcome category.

However, two other instances of the zero cell count problem are likely to occur in swallowing outcomes research and these may pose challenges to the suitability of the multinomial logistic regression approach. First, for the current data set, we stated an expectation that all participants were included in the trial on the basis of demonstrating a worst baseline PAS score of 3 or higher. In using multinomial logistic regression with the example data set, we determined that the absence of PAS scores in the healthy range (i.e., 1 and 2) at baseline led to a failure of model convergence assuming a repeated measures design with both pre- and post-treatment data. A similar dilemma might well have occurred in the case that no observations of PAS = 8 were present for a given combination of Consistency and Group in the post-treatment data: the model would fail to converge. In other words, if a treatment is so good that no participant continues to display silent aspiration at the post-treatment assessment, the model will not be able to calculate the probability of silent aspiration in that treatment condition. The *zero cell count* can occur in binary logistic regression as well, but it becomes more likely in multinomial models because there are so many individual outcome categories.

##### Ordinal Logistic Regression

In the introduction, we discussed historical survey data that suggest that some clinicians are uncertain whether the 8 categories of the PAS are correctly ordered as numerated [6]. Nevertheless, we have proposed that the PAS could be re-ordered or collapsed into categories that are ordered based on a physiological framework, as suggested in the proposed Categorical PAS with possible labels of A, B, C, and D. If one accepts that category A is less severe than category B and so forth, ordinal logistic regression becomes a suitable approach to analysis. In our opinion, this is not only the most appropriate approach to analysis of PAS data, but it will also provide the richest information with respect to differences in airway protection status. Furthermore, this approach has the advantage of reduced complexity for interpretation compared to the multinomial approach described above.

There are a number of versions of ordinal logistic regression. For the purposes of illustration, we will describe the most common approach, the Proportional Odds Model [69, 71–73], which is available in most statistical software packages (e.g., SPSS PLUM, SAS proc logistic, Stata’s ologit). Here, the equation is written as follows:

$${\text{Ln}}\left( {{\text{Odds}}_{j} } \right){\mkern 1mu} = \theta j - {\mkern 1mu} (\beta _{0} + \beta _{1} X_{1} + \beta _{2} X_{2} + {\mkern 1mu} \ldots {\mkern 1mu} + \beta _{{kXk}} + \varepsilon _{i} ).$$

1. Here, the intercept *β*_*0*_ has been replaced by a new function, θ*j*, where *j* represents the number of ordered categories. This intercept represents a threshold value in the model, at which the odds shifts from the dependent variable being in a lower-ordered category into the next higher-ordered category. An important aspect of this approach is the proportional odds assumption, which states that the difference in odds for different categories in the model lies in this threshold value. Consequently each outcome category has its own intercept, but all outcome categories share the same regression coefficient.

Remember that in the multinomial model, the odds of each category are compared to a single reference category. Here, we measure the odds of *any* lower category in comparison to *any* higher category.

The calculation of unique intercepts allows for the fact that some outcome categories are simply more frequent than others, while the single regression coefficient measures how the independent variables relate to the odds of being in *any* lower category. It says, for example, that the effect of using a thicker bolus consistency is generalized in the sense that it has the same effect on the odds of a PAS score = 7 compared to a PAS score = 8 as it does on the odds of a PAS score = 5 compared to PAS scores of either 7 or 8. This assumption is important to understand but is often violated in real data and needs to be checked. Table 6, below, provides frequency data for the illustrative dataset with PAS scores collapsed according to the scheme suggested above.

**Table 6:** Frequencies and percentages for categorical PAS scores in the hypothetical dataset by treatment group and consistency

Group	Consistency	Statistic	PASCAT = A	PASCAT = B	PASCAT = C	PASCAT = D
Control	Thin	Count	19	13	5	3
		%	47.5%	32.5%	13%	7%
	Mildly thick	Count	20	11	3	6
		%	50%	28%	7%	15%
Experimental	Thin	Count	23	9	3	5
		%	57.5%	22.5%	7%	13%
	Mildly thick	Count	33	4	2	1
		%	82.5%	10%	5%	2.5%

Table 7 provides regression coefficients (intercepts) and odds ratios for the Ordinal PAS categories of A vs. B, C, and D; A or B vs. C and D; and A, B or C vs D, respectively, given factors of group (experimental vs. control) and consistency (mildly thick vs. thin) and their interaction. The threshold coefficients are not usually interpreted individually, but they represent the intercepts, specifically the point (in terms of a logit or log-odds function) where PAS scores can be predicted to shift into higher-ordered categories. The odds ratios for the effects of group and consistency and their interaction tell us how these predictors affect the odds of a swallow having a penetration-aspiration score in a higher-ordered category. Table 8 provides greater detail regarding the interaction, showing that being in the experimental group has a stronger effect on the odds of a seeing a higher-ordered (i.e., less severe) PAS category with the mildly thick liquid compared to the thin liquid.

**Table 7:** Output for ordinal logistic regression analysis of treatment group differences in categorical PAS scores by consistency

Comparison	Threshold (intercept)	Standard error	Wald *χ* ^2^	Significance	Odds ratio
PAS category A vs. categories B, C and D	0.251	0.309	0.661	0.416	1.286
PAS categories A or B vs. categories C and D	1.485	0.337	19.479	0.000	4.417
PAS categories A, B or C vs. category D	2.218	0.379	34.172	0.000	9.192
Group × consistency	−1.308	0.664	3.887	0.049	0.270
Group (experimental vs. control)	0.248	0.426	0.339	0.561	1.282
Consistency (thin vs. mildly thick)	0.025	0.419	0.003	0.953	1.025

**Table 8:** Post-hoc output for ordinal logistic regression analysis comparing the odds of different categorical PAS scores for the interaction of experimental group plus mildly thick liquids

Comparison	Threshold (intercept)	Standard error	Wald *χ* ^2^	Significance	Odds ratio
PAS category A vs. categories B, C and D	0.003	0.303	0.000	0.991	1.003
PAS categories A or B vs. categories C and D	1.237	0.324	14.569	0.000	3.447
PAS categories A, B or C vs. category D	1.970	0.266	28.915	0.000	7.173
Group × consistency	−1.308	0.664	3.887	0.049	0.780
Group (control vs. experimental)	−0.248	0.426	0.339	0.561	1.025
Consistency (mildly thick vs. thin)	0.025	0.419	0.003	0.953	0.270

## Conclusions

In this article, we have outlined an argument for treating the Penetration-Aspiration Scale as categorical, with the possibility of ordered categories given physiological considerations. The statistical sections of this paper outline the flaws of treating the PAS as an interval scale and propose that frequency distributions, odds ratios, and logistic regression models are more appropriate and powerful solutions for the analysis of PAS data. Although using the ordinal logistic regression model on an appropriate ordering of the PAS data may lead to similar conclusions as the earlier inappropriate treatment of the data as interval, this result is achieved without the need to make untenable assumptions and allows confident interpretations in terms of the probability of witnessing each ordered category, which makes sense in this situation. Given that ordinal logistic regression procedures are readily available in mainstream software, and include the option to incorporate covariates, interactions, and repeated measures into the model, we strongly encourage their use for future analysis of PAS data.

## Appendix

See Table 9.

**Table 9:** Hypothetical individual participant worst PAS scores at a post-treatment videofluoroscopy for thin and mildly thick consistencies, organized by treatment group: experimental (E) and control (C)

Participant	PAS score (thin)	PAS score (mildly thick)	Participant	PAS score (thin)	PAS score (mildly thick)
E-1	5	1	C-1	5	8
E-2	1	1	C-2	5	8
E-3	1	1	C-3	1	8
E-4	1	1	C-4	1	2
E-5	1	1	C-5	1	3
E-6	8	1	C-6	5	1
E-7	1	1	C-7	7	8
E-8	5	2	C-8	1	5
E-9	1	1	C-9	1	7
E-10	1	1	C-10	8	8
E-11	1	2	C-11	1	1
E-12	1	1	C-12	2	1
E-13	2	1	C-13	7	8
E-14	5	5	C-14	1	3
E-15	2	1	C-15	1	1
E-16	1	1	C-16	1	1
E-17	8	1	C-17	1	2
E-18	3	1	C-18	1	5
E-19	8	2	C-19	3	3
E-20	1	1	C-20	3	5
E-21	3	2	C-21	5	1
E-22	3	2	C-22	5	2
E-23	3	2	C-23	2	1
E-24	3	1	C-24	3	1
E-25	1	2	C-25	1	1
E-26	1	7	C-26	3	3
E-27	5	7	C-27	2	1
E-28	1	1	C-28	2	1
E-29	1	2	C-29	5	1
E-30	7	2	C-30	2	3
E-31	2	8	C-31	3	5
E-32	2	1	C-32	2	1
E-33	2	1	C-33	2	1
E-34	2	2	C-34	3	2
E-35	2	2	C-35	7	2
E-36	7	3	C-36	7	2
E-37	7	3	C-37	8	3
E-38	8	3	C-38	8	3
E-39	8	1	C-39	3	7
E-40	2	2	C-40	7	7

## References

[1] Rosenbek JC, Robbins JA, Roecker EB, Coyle JL, Wood JL (1996). A penetration-aspiration scale. Dysphagia.

[2] Colodny N (2002). Interjudge and intrajudge reliabilities in fiberoptic endoscopic evaluation of swallowing (FEES) using the penetration-aspiration scale: a replication study. Dysphagia.

[3] Kelly AM, Drinnan MJ, Leslie P (2007). Assessing penetration and aspiration: how do videofluoroscopy and fiberoptic endoscopic evaluation of swallowing compare?. Laryngoscope.

[4] Rao N, Brady SL, Chaudhuri G, Donzelli JJ, Wesling MW (2003). Gold-Standard? Analysis of the videofluoroscopic and fiberoptic endoscopic swallow examinations. J Appl Res Clin Exp Ther.

[5] Butler SG, Stuart A, Case LD, Rees C, Vitolins M, Kritchevsky SB (2011). Effects of liquid type, delivery method, and bolus volume on penetration-aspiration scores in healthy older adults during flexible endoscopic evaluation of swallowing. Ann Otol Rhinol Laryngol.

[6] McCullough GH, Rosenbek JC (1998). Ordinality and intervality of a penetration-aspiration scale. J Med Speech Lang Pathol.

[7] Grace-Martin K. When a variable’s level of measurement isn’t obvious. 2014. http://www.theanalysisfactor.com/level-of-measurement-not-obvious/. Accessed 11 Apr 2017.

[8] Langmore SE, McCulloch TM, Krisciunas GP, Lazarus CL, Van Daele DJ, Pauloski BR, Rybin D, Doros G (2016). Efficacy of electrical stimulation and exercise for dysphagia in patients with head and neck cancer: a randomized clinical trial. Head Neck.

[9] Troche MS, Okun MS, Rosenbek JC, Musson N, Fernandez HH, Rodriguez R, Romrell J, Pitts T, Wheeler-Hegland KM, Sapienza CM (2010). Aspiration and swallowing in Parkinson disease and rehabilitation with EMST: a randomized trial. Neurology.

[10] Pitts T, Bolser D, Rosenbek J, Troche M, Okun MS, Sapienza C (2009). Impact of expiratory muscle strength training on voluntary cough and swallow function in Parkinson disease. Chest.

[11] Robbins J, Coyle J, Rosenbek J, Roecker E, Wood J (1999). Differentiation of normal and abnormal airway protection during swallowing using the penetration-aspiration scale. Dysphagia.

[12] Robbins J, Kays SA, Gangnon RE, Hind JA, Hewitt AL, Gentry LR, Taylor AJ (2007). The effects of lingual exercise in stroke patients with dysphagia. Arch Phys Med Rehab.

[13] Pearson WG, Davidoff AA, Smith ZM, Adams DE, Langmore SE (2016). Impaired swallowing mechanics of post radiation therapy head and neck cancer patients: a retrospective videofluoroscopic study. World J Radiol.

[14] Malandraki GA, Rajappa A, Kantarcigil C, Wagner E, Ivey C, Youse K (2016). The intensive Dysphagia rehabilitation approach applied to patients with neurogenic Dysphagia: a case series design study. Arch Phys Med Rehab.

[15] Power ML, Hamdy S, Goulermas JY, Tyrrell PJ, Turnbull I, Thompson DG (2009). Predicting aspiration after hemispheric stroke from timing measures of oropharyngeal bolus flow and laryngeal closure. Dysphagia.

[16] Argolo N, Sampaio M, Pinho P, Melo A, Nobrega AC (2015). Videofluoroscopic predictors of penetration-aspiration in Parkinson’s disease patients. Dysphagia.

[17] Steele CM, Bailey GL, Polacco RE, Hori SF, Molfenter SM, Oshalla M, Yeates EM (2013). Outcomes of tongue-pressure strength and accuracy training for dysphagia following acquired brain injury. Int J Speech Lang Pathol.

[18] Steele CM, Bayley MT, Peladeau-Pigeon M, Nagy A, Namasivayam AM, Stokely SL, Wolkin T (2016). A randomized trial comparing two tongue-pressure resistance training protocols for post-stroke Dysphagia. Dysphagia.

[19] Martin-Harris B, McFarland D, Hill EG, Strange CB, Focht KL, Wan Z, Blair J, McGrattan K (2015). Respiratory-swallow training in patients with head and neck cancer. Arch Phys Med Rehab.

[20] Plowman EK, Watts SA, Robison R, Tabor L, Dion C, Gaziano J, Vu T, Gooch C (2016). Voluntary cough airflow differentiates safe versus unsafe swallowing in amyotrophic lateral sclerosis. Dysphagia.

[21] Michou E, Mistry S, Jefferson S, Singh S, Rothwell J, Hamdy S (2012). Targeting unlesioned pharyngeal motor cortex improves swallowing in healthy individuals and after dysphagic stroke. Gastroenterology.

[22] Starmer HM, Quon H, Kumar R, Alcorn S, Murano E, Jones B, Humbert I (2015). The effect of radiation dose on swallowing: evaluation of aspiration and kinematics. Dysphagia.

[23] Pikus L, Levine MS, Yang YX, Rubesin SE, Katzka DA, Laufer I, Gefter WB (2003). Videofluoroscopic studies of swallowing dysfunction and the relative risk of pneumonia. AJR Am J Roentgenol.

[24] Doggett DL, Tappe KA, Mitchell MD, Chapell R, Coates V, Turkelson CM (2001). Prevention of pneumonia in elderly stroke patients by systematic diagnosis and treatment of dysphagia: an evidence-based comprehensive analysis of the literature. Dysphagia.

[25] Ribeiro PW, Cola PC, Gatto AR, da Silva RG, Luvizutto GJ, Braga GP, Schelp AO, de Arruda Henry MA, Bazan R (2015). Relationship between Dysphagia, national institutes of health stroke scale score, and predictors of pneumonia after ischemic stroke. J Stroke Cerebrovasc Dis.

[26] Langmore SE, Terpenning MS, Schork A, Chen Y, Murray JT, Lopatin D, Loesche W (1998). Predictors of aspiration pneumonia: how important is Dysphagia?. Dysphagia.

[27] Silver KH, Van Nostrand D (1994). The use of scintigraphy in the management of patients with pulmonary aspiration. Dysphagia.

[28] Albeldawi M, Makkar R (2012). Images in clinical medicine. Barium aspiration. N Engl J Med.

[29] Marik PE (2001). Aspiration pneumonitis and aspiration pneumonia. N Engl J Med.

[30] Scannapieco FA (1999). Role of oral bacteria in respiratory infection. J Periodontol.

[31] Scannapieco FA, Rethman MP (2003). The relationship between periodontal diseases and respiratory diseases. Dent Today.

[32] Brogan E, Langdon C, Brookes K, Budgeon C, Blacker D (2014). Respiratory infections in acute stroke: nasogastric tubes and immobility are stronger predictors than dysphagia. Dysphagia.

[33] Langmore SE (1999). Risk factors for aspiration Pneumonia. Nutr Clin Pract.

[34] Langmore SE, Skarupski KA, Park PS, Fries BE (2002). Predictors of aspiration pneumonia in nursing home residents. Dysphagia.

[35] Terpenning M (2005). Geriatric oral health and pneumonia risk. Clin Infect Dis.

[36] Robbins J, Hamilton JW, Lof GL, Kempster GB (1992). Oropharyngeal swallowing in normal adults of different ages. Gastroenterology.

[37] Kendall KA, Leonard RJ (2001). Bolus transit and airway protection coordination in older dysphagic patients. Laryngoscope.

[38] Kendall KA, Leonard RJ, McKenzie S (2004). Airway protection: evaluation with videofluoroscopy. Dysphagia.

[39] Daggett A, Logemann J, Rademaker A, Pauloski B (2006). Laryngeal penetration during deglutition in normal subjects of various ages. Dysphagia.

[40] Jean A (2001). Brain stem control of swallowing: neuronal network and cellular mechanisms. Physiol Rev.

[41] Steele CM, Miller AJ (2010). Sensory input pathways and mechanisms in swallowing: a review. Dysphagia.

[42] Ludlow CL (2015). Laryngeal reflexes: physiology, technique, and clinical use. J Clin Neurophysiol.

[43] Lang IM (2009). Brain stem control of the phases of swallowing. Dysphagia.

[44] Miller A, Bieger D, Conklin J, Perlman AL, Schulze-Delrieu KS (1997). Functional controls of deglutition. Deglutition and its Disorders.

[45] Miller AJ (1972). Significance of sensory inflow to the swallowing reflex. Brain Res.

[46] Shaker R (1993). Functional relationship of the larynx and upper GI tract. Dysphagia.

[47] Shaker R, Dodds WJ, Dantas RO, Hogan WJ, Arndorfer RC (1990). Coordination of deglutitive glottic closure with oropharyngeal swallowing. Gastroenterology.

[48] Shaker R, Hogan WJ (2000). Reflex-mediated enhancement of airway protective mechanisms. Am J Med.

[49] Martin-Harris B, Brodsky MB, Michel Y, Castell DO, Schleicher M, Sandidge J, Maxwell R, Blair J (2008). MBS measurement tool for swallow impairment–MBSImp: establishing a standard. Dysphagia.

[50] Molfenter SM, Steele CM (2014). Kinematic and temporal factors associated with penetration-aspiration in swallowing liquids. Dysphagia.

[51] Troche MS, Brandimore AE, Okun MS, Davenport PW, Hegland KW (2014). Decreased cough sensitivity and aspiration in Parkinson disease. Chest.

[52] Bath PM, Scutt P, Love J, Clave P, Cohen D, Dziewas R, Iversen HK, Ledl C, Ragab S, Soda H, Warusevitane A, Woisard V, Hamdy S (2016). Swallowing treatment using pharyngeal electrical stimulation trial I: pharyngeal electrical stimulation for treatment of Dysphagia in subacute stroke: a randomized controlled trial. Stroke.

[53] Westfall P, Henning KSS (2013). Understanding advanced statistical methods.

[54] Logemann JA, Gensler G, Robbins J, Lindblad AS, Brandt D, Hind JA, Kosek S, Dikeman K, Kazandjian M, Gramigna GD, Lundy D, McGarvey-Toler S, Miller Gardner PJ (2008). A randomized study of three interventions for aspiration of thin liquids in patients with dementia or Parkinson’s disease. J Speech Lang Hearing Res.

[55] Rofes L, Arreola V, Mukherjee R, Swanson J, Clave P (2014). The effects of a xanthan gum-based thickener on the swallowing function of patients with dysphagia. Aliment Pharmacol Ther.

[56] Leonard RJ, White C, McKenzie S, Belafsky PC (2014). Effects of bolus rheology on aspiration in patients with Dysphagia. J Acad Nutr Diet.

[57] Clave P, de Kraa M, Arreola V, Girvent M, Farre R, Palomera E, Serra-Prat M (2006). The effect of bolus viscosity on swallowing function in neurogenic dysphagia. Aliment Pharmacol Ther.

[58] Rasley A, Logemann JA, Kahrilas PJ, Rademaker AW, Pauloski BR, Dodds WJ (1993). Prevention of barium aspiration during videofluoroscopic swallowing studies: value of change in posture. AJR Am J Roentgenol.

[59] Shanahan TK, Logemann JA, Rademaker AW, Pauloski BR, Kahrilas PJ (1993). Chin-down posture effect on aspiration in dysphagic patients. Arch Phys Med Rehab.

[60] Macrae P, Anderson C, Humbert I (2014). Mechanisms of airway protection during chin-down swallowing. J Speech Lang Hearing Res.

[61] Fink TA, Ross JB (2009). Are we testing a true thin liquid?. Dysphagia.

[62] Plowman EK, Tabor LC, Robison R, Gaziano J, Dion C, Watts SA, Vu T, Gooch C (2016). Discriminant ability of the Eating Assessment Tool-10 to detect aspiration in individuals with amyotrophic lateral sclerosis. Neurogastroenterol Motil.

[63] Hind JA, Gensler G, Brandt DK, Gardner PJ, Blumenthal L, Gramigna GD, Kosek S, Lundy D, McGarvey-Toler S, Rockafellow S, Sullivan PA, Villa M, Gill GD, Lindblad AS, Logemann JA, Robbins J (2009). Comparison of trained clinician ratings with expert ratings of aspiration on videofluoroscopic images from a randomized clinical trial. Dysphagia.

[64] Molfenter SM, Steele CM (2013). The relationship between residue and aspiration on the subsequent swallow: an application of the normalized residue ratio scale. Dysphagia.

[65] Bonilha HS, Blair J, Carnes B, Huda W, Humphries K, McGrattan K, Michel Y, Martin-Harris B (2013). Preliminary investigation of the effect of pulse rate on judgments of swallowing impairment and treatment recommendations. Dysphagia.

[66] Langmore SE, Schatz K, Olsen N (1988). Fiberoptic endoscopic examination of swallowing safety: a new procedure. Dysphagia.

[67] Conover WJ, Iman RL (1981). Rank transformations as a bridge between parametric and nonparametric statistics. Am Stat.

[68] Zimmerman DW, Zumbo BD (1993). Relative power of the Wilcoxon test, the Friedman test, and repeated measures ANOVA on ranks. J Exp Educ.

[69] Fox J, Andersen R (2006). Effect displays for multinomial and proportional-odds logit models. Sociol Methodol.

[70] Grace-Martin K. Binary, ordinal, and multinomial logistic regression for categorical outcomes. http://www.theanalysisfactor.com/binary-ordinal-multinomial-logistic/. Accessed 15 May 2017.

[71] Long JS, Freese J. Regression models for categorical variables with stata. college station, TX: Stata Press, 2001. http://stats.idre.ucla.edu/stata/webbooks/reg/chapter3/regression-with-statachapter-3-regression-with-categorical-predictors/. Accessed 11 April 2017.

[72] Agresti A. Modeling ordinal categorical data. 2010. http://www.stat.ufl.edu/~aa/ordinal/agresti_ordinal_tutorial.pdf Accessed 11 April 2017.

[73] Agresti A (1996). An introduction to categorical data analysis.

